# Investigation on hydrodynamic lubrication effect of micro groove seal in pharmaceutical kettle

**DOI:** 10.1371/journal.pone.0291360

**Published:** 2023-09-11

**Authors:** Yinghua Zhou, Xing Cheng, Fengming Sun, Ran Gong

**Affiliations:** 1 School of Medicine, Jiangsu University, Zhenjiang, Jiangsu, China; 2 School of Automotive and Traffic Engineering, Jiangsu University, Zhenjiang, Jiangsu, China; National Kaohsiung University of Science and Technology / Industrial University of Ho Chi Minh, TAIWAN

## Abstract

To improve the lubrication conditions of the seal in the pharmaceutical kettles, a specific shape groove with micrometer level on the sealing end face is set up to fully utilize the fluid dynamic pressure effect under given working conditions. A numerical model is developed to solve the pressure distribution in the micro groove, where any groove shape can be used. The numerical form of the model is derived using the principle of mass conservation without considering the film thickness derivative term, and the coordinate transformation is introduced to adapt to the curved shape of the groove. The cavitation phenomenon is taken into account in the flow field of the seal, and the JFO cavitation model is introduced to modify the Reynolds equation. The diversity of groove shapes is considered, and the node adsorption method is adopted to approximate the groove shape. The model is established based on the principle of mass conservation, which can adapt to any different groove shapes and has a strong scalability. By mathematical modeling and solving, the performances of the micro groove seal under different groove shapes are analyzed, providing a basis for the micro groove design of seal in pharmaceutical kettles.

## 1. Introduction

Stirred kettle reactors are widely used in chemical, food, biological, papermaking, pharmaceutical, and petroleum industries as equipment for physical and chemical reactions [1–3]. The precondition for the functions of hydrogenation, mixing, hydrolysis, crystallization, and other reactions in the kettle is the sufficient stirring and mixing of the substances participating in the kettle. In pharmaceutical kettles, the seal between the stirring shaft and the shell of the stirred kettle was previously filled with packing seal, which resulted in significant leakage and energy loss, seriously affecting the continuity of the process and production efficiency. With the continuous development of mechanical seal technology, mechanical seals have completely replaced packing seals as the main seals between the stirring rotating shaft and the kettle body in important production processes.

Based on previous statistical results of failure causes, it is found that most rotating shaft equipment failures are due to mechanical seal failure [4]. Therefore, the performance of mechanical seals is crucial for the continuous operation of rotating shaft equipment. With the deepening research on seals, the oil film status on the end face of the seal has become a noteworthy research topic [5–8]. The oil film status on the end face of the seal is not only the source of leakage but also the necessary lubricant, and more and more researchers have begun to conduct corresponding research on it. Salant et al. [9] studied the lubrication status of the sealing end face from a theoretical analysis perspective. Some scholars have considered the influence of the roughness of the sealing end face and believed that the oil film thickness of the end face is basically at the same level as the seal surface roughness [10–12]. When the rotating sealing surface is in a mixed lubrication state, the radial cone of high-frequency roughness, low-frequency waviness, and overall shape error in the surface morphology have a significant impact on the friction performance of the sealing surface. These studies further demonstrate the complexity of sealing face design in rotating machinery.

**Fig 1:**
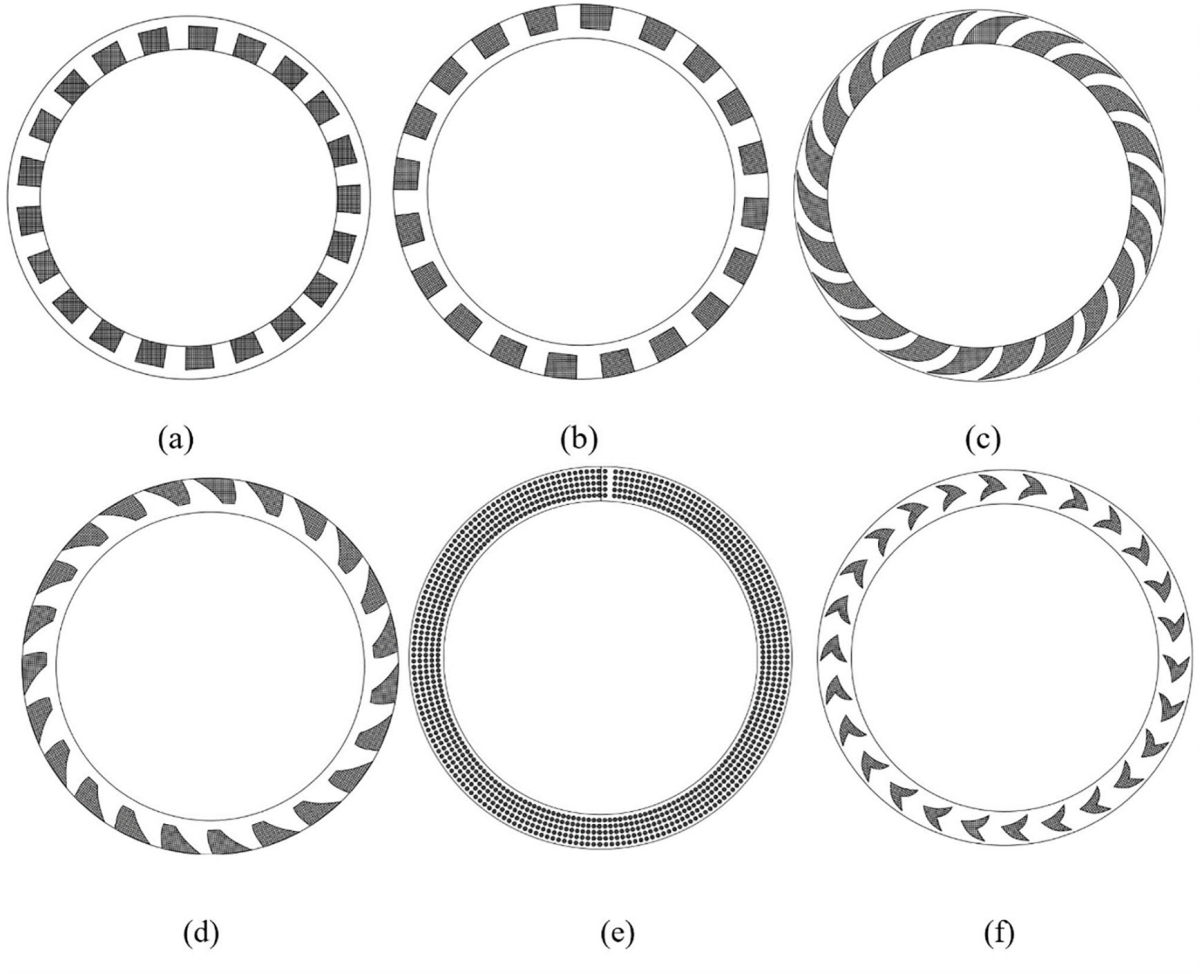
Some common seal grooves. (a): Inner rectangular groove. (b): Outer rectangular groove. (c): Spiral groove. (d): Spiral shaped non through groove. (e): Micro textured hole. (f): Dovetail groove.

Under certain speed and pressure conditions, conventional seal cannot provide effective sealing and lubrication due to various reasons. As a result, the sealing end face cannot be in a hydrodynamic lubrication state, and the seal experiences severe friction and wear, easily leading to seal failure. Experimental studies have shown that the rotating sealing end face can only operate in the fluid dynamic lubrication region under certain conditions [13, 14]. Inspired by the fluid dynamic pressure and hydrostatic bearings, scholars have actively used the method of grooving (including grooves, steps, slopes, and holes) in the rotating sealing end face to improve the lubrication status of the end face by utilizing fluid dynamic and hydrostatic effects. The grooving on the end face can be roughly divided into two categories: deep grooves and shallow grooves according to the groove depth. Some common groove types of seals are shown in Fig 1.

Shallow grooves refer to various types of grooves with a depth of micrometers, which mainly rely on the fluid dynamic pressure effect to establish dynamic pressure on the sealing end face and promote film formation to achieve non-contact of the sealing face. Yu et al. [15] obtained the average hydrodynamic pressures for circular, elliptic and triangular textures with different sliding directions. Shen et al. [16] used a sequential quadratic programming to determine the optimum texture shape to generate higher load-carrying capacity. It was found that the chevron-shapes with flat fronts was the optimum shape under unidirectional sliding, while the optimum texture for bidirectional sliding was a pair of trapezoid-like shapes. The basis for the seal groove design method comes from the Whipple bearing theory. The control equation for the fluid film of the end face seal is the Reynolds equation. When the fluid film thickness is continuous, the Reynolds equation can be directly discretized, such as micro pit surfaces, wavy surfaces, or wedge-shaped surfaces [7, 17–19]. Khonsari’s group used the half-Sommerfeld boundary model to solve the Reynolds equation [20]. Shen and Khonsari [21] supposed that the error caused by the half-Sommerfeld boundary condition was about 5% compared to that of the Jacobsson-Floberg-Olsson (JFO) boundary model.

In the process of operation of high-speed centrifugation kettles in the field of biomedical pharmaceuticals, it is required to ensure the complete isolation of the extraction channel from the external environment while also ensuring a longer lifespan of the sealing ring. The method of grooving on the sealing face is adopted to actively utilize the fluid dynamic pressure effect to improve the lubrication state of the sealing end face. Further improvement of the fluid dynamic pressure effect of the seal and the enhancement of the lubrication status of the sealing end face have created new possibilities for the development of hydrodynamic type mechanical seals. The measures such as grooving and hole punching can be implemented on the end face of the seal to satisfy the conditions for generating hydrodynamic pressure. In this paper, the seal in a single end pharmaceutical kettle is studied, and the discrete form suitable for numerical calculation is derived from the Reynolds equation. The JFO cavitation condition is considered, and finite volume method is used for discretization. The Gauss-Siedel method is used to solve the self-programmed numerical equation, obtaining a structured numerical solution method for the seal gap flow field that varies with the structural and operating parameters of the seal. The performance differences and their expressions among different groove types are analyzed, which provides a theoretical basis for the groove design of the seal.

## 2. Flow field model of micro groove seal

**Fig 2:**
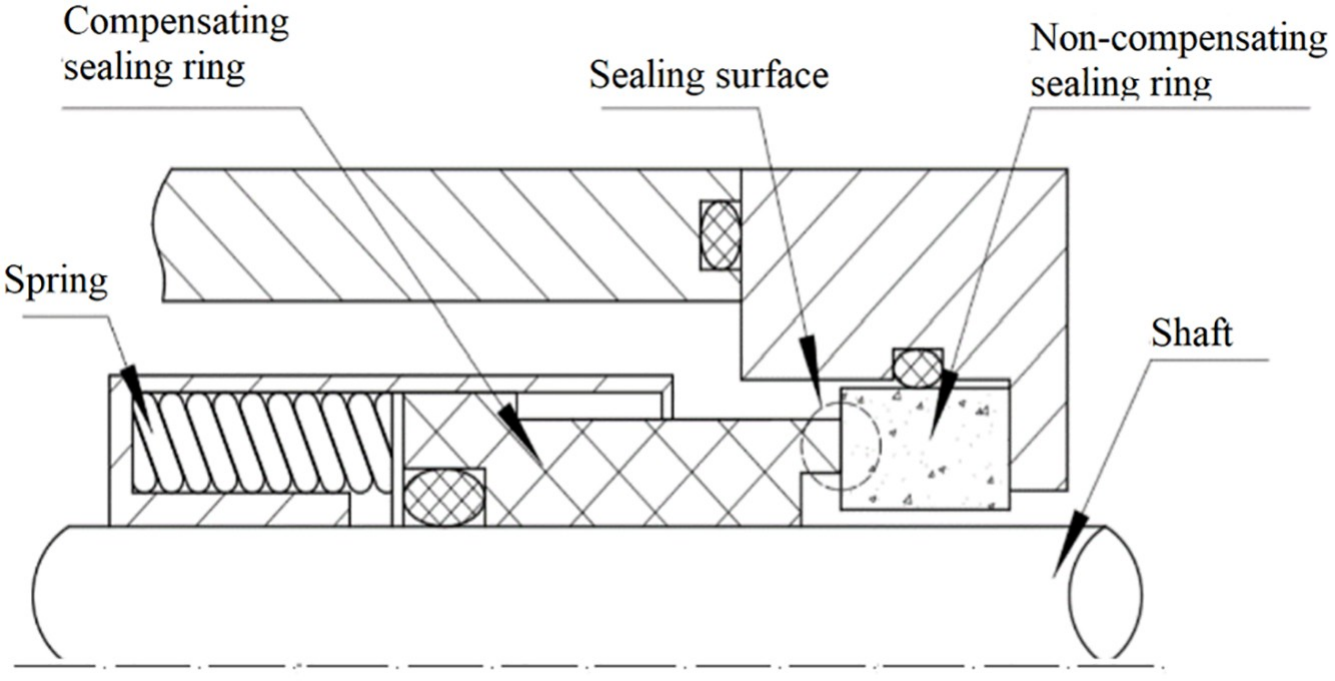
Structure of mechanical seal in pharmaceutical kettle.

Ideally, the lubrication status of the end face of the seal should exhibit a complete transition from dry friction to full fluid film lubrication, and eventually stabilize in a full fluid film lubrication state. However, due to the structural limitations of the seal itself and the changes in complex working conditions, the seal cannot maintain a stable full fluid film lubrication state and exhibits characteristics of mixed friction. The geometric structure of the mechanical seal is shown in Fig 2. As a common form of rotating shaft dynamic seal, mechanical seals are devices that prevent fluid leakage by maintaining contact and relative sliding between at least one pair of end faces perpendicular to the axis under the action of fluid pressure and compensating mechanism elasticity, as well as auxiliary seal. The seal is installed to slide and fit by the spring force and medium pressure. The structure of the mechanical seal varies, but the basic form consists of the following parts: the sealing ring, which is composed of a compensating ring and a non-compensating ring for compensation; an elastic element that provides spring force compensation; an auxiliary sealing ring. In this study, the outer diameter of the sealing ring is 130 mm, the inner diameter is 117.2 mm, and the sealing material is silicon carbide. The mechanical seals for kettles generally involve variable speed and pressure. The commonly used speed working condition is 500–2000 r/min, and the pressure is 0.5–2 MPa. The external sealing fluid system uses ethylene glycol as the sealing fluid.

In order to establish an analysis model for the fluid film on the sealing face, the following assumptions are made.

1. The sealed fluid is Newtonian fluid without considering the effect of turbulence on the flow field.
2. The sealing ring is rigid body, and the surface does not deform.
3. The thickness of the fluid film on the sealing face is in the micrometer level, and it is believed that the film pressure is fixed along the thickness direction.
4. The sealing ring does not tilt, twist or deform during operation, and therefore it is believed that the thickness of the film does not change after stability.

Use Reynolds equation to describe the flow field motion in the gap of the sealing ring. The Reynolds equation for ideal fluid stability in cylindrical coordinates under isothermal conditions can be described as [22]

$$\frac{1}{r}\frac{\partial }{\partial r}\left(\frac{\rho rh^{3}}{\mu }\frac{\partial p}{\partial r}\right)+\frac{1}{r}\frac{\partial }{\partial \theta }\left(\frac{\rho h^{3}}{r\mu }\frac{\partial p}{\partial \theta }\right)=6\omega \frac{\partial \left(\rho h\right)}{\partial \theta }+\frac{3\omega^{2}}{10r}\frac{\partial }{\partial r}\left(\frac{\rho^{2}r^{2}h^{3}}{\mu }\right)$$ (1)

where *h* is the fluid film thickness, *μ* is the dynamic viscosity of the lubricating oil, *p* is the fluid film pressure, *ω = ω*_*s*_*-ω*_*r*_ is the relative rotational angular velocity, and *ω*_*s*_ and *ω*_*r*_ represent the rotational angular velocity of the rotating shaft and seal respectively.

Since the equation contains multiple variables, it is necessary to first determine the solving variables. The research focus of this paper is on the evaluation parameter of fluid film pressure distribution, so pressure *p* is used as the solution parameter, while other variables such as density *ρ*, the film thickness *h* needs to be determined in advance or simplified accordingly.

The fluid pressure at the inlet and outlet of the seal is set to be constant, so the boundary conditions for the fluid film control equation are

$$\left\{\begin{array}{c} r=r_{i},\;p=p_{i}\\ r=r_{0},\;p=p_{0} \end{array}\right.$$ (2)

That is, the pressure of inner and outer diameters is constant, which belongs to the first kind of boundary condition (Dirichlet boundary condition).

Further considering the actual structure of the sealing ring, the groove shape exhibits a periodic distribution. In the actual calculation process, only one period can be selected as the calculation period, thereby adding periodic boundary conditions to the mathematical model

$$\left\{\begin{array}{l} p{\left.\right|}_{\theta }=p{\left.\right|}_{\theta +\frac{2\pi }{N_{g}}}\\ \frac{\partial p}{\partial \theta }{\left.\right|}_{\theta }=\frac{\partial p}{\partial \theta }{\left.\right|}_{\theta +\frac{2\pi }{N_{g}}} \end{array}\right.$$ (3)

where *N*_*g*_ is the number of grooves on the sealing ring, and *θ* represents any angle, and $0\leq \theta <\frac{2\pi }{N_{g}}$.

## 3. Cavitation effects

When fluid flows through seal gaps, uneven pressure distribution along the circumference of the gap is caused by the effect of the groove shape. In some areas of the groove shape edge, pressure can be so low that oil density decreases, leading to a mixture of oil and gas known as cavitation phenomenon in the fluid. Cavitation occurrence can lead to incorrect simulation of the flow field and results in reduced computing accuracy and lateral offset of the calculated pressure distribution compared to the correct results. In order to improve the accuracy and precision of pressure distribution calculation, it is necessary to consider further the density changes in the fluid and to handle the cavitation area separately, thereby including its impact in the model. This paper uses the improved JFO cavitation boundary condition to simulate the cavitation phenomenon in seal gap flow.

The ratio of fluid film density *ρ* to sealing medium density *ρ*_L_ is denoted by *θ*. When considering the influence of cavitation, the control equation of the flow field can be described as follows.


$$\left\{\begin{array}{c} \frac{1}{r}\frac{\partial }{\partial r}\left(\frac{rh^{3}}{\mu }\frac{\partial p}{\partial r}\right)+\frac{1}{r}\frac{\partial }{\partial \theta }\left(\frac{h^{3}}{r\mu }\frac{\partial p}{\partial \theta }\right)=6\omega \frac{\partial \left(h\right)}{\partial \theta }+\frac{3\omega^{2}}{10r}\frac{\partial }{\partial r}\left(\frac{\rho r^{2}h^{3}}{\mu }\right)p>p_{c},\rho =\rho_{L}\\ 0=2\frac{\partial \left(\rho h\right)}{\partial \theta }+\frac{\omega }{10r}\frac{\partial }{\partial r}\left(\frac{\rho^{2}r^{2}h^{3}}{\mu }\right)p=p_{c},\rho <\rho_{L}\;(\text{Cavitaiton}\;\text{area}) \end{array}\right.$$ (4)

The definition of the general variable *φ* and the switching function *F* is as follows

$$\frac{p-p_{c}}{p_{a}-p_{c}}=F\varphi$$ (5)

$$\frac{\rho }{\rho_{c}}=1+\left(1-F\right)\varphi$$ (6)

$$\left\{\begin{array}{c} \varphi \geq 0,F=1\\ \varphi <0,F=0 \end{array}\right.$$ (7)

Adjust the above equation to obtain *p* and *ρ* replacement equation

$$\left\{\begin{array}{c} p=F\varphi \left(p_{a}-p_{c}\right)+p_{c}\\ \rho =\left(1+\left(1-F\right)\varphi \right)\rho_{c} \end{array}\right.$$ (8)

where *p*_*a*_ is standard atmospheric pressure, *p*_*c*_ is the cavitation pressure, and *ρ*_*c*_ is the fluid density.

Therefore, the control equation is modified to

$$\begin{array}{c} \frac{\partial }{\partial r}\left(-\frac{\left(1+\left(1-F\right)\varphi \right)rh^{3}}{\mu }\frac{\partial F\varphi }{\partial r}\right)+\frac{1}{r}\frac{\partial }{\partial \theta }\left(-\frac{\left(1+\left(1-F\right)\varphi \right)h^{3}}{r\mu }\frac{\partial F\varphi }{\partial \theta }\right)+\\ 6\omega \frac{\partial }{\partial \theta }\left(1+\left(1-F\right)\varphi \right)+\frac{\frac{3\omega^{2}}{10r}\frac{\partial }{\partial r}{\left(\left(1+\left(1-F\right)\right)\varphi \right)}^{2}\rho_{c}\left(r^{2}h^{3}\right)}{\mu }=0 \end{array}$$ (9)

Further dimensionless, adjust the control equation to

$$\begin{array}{c} \frac{\partial }{\partial \bar{r}}\left(-\bar{h^{3}}\frac{\partial F\varphi }{\partial \bar{r}}\right)+\frac{\partial }{\theta_{0}^{2}\partial \bar{\theta }}\left(-\bar{h^{3}}\frac{\partial F\varphi }{\bar{r}\partial \bar{\theta }}\right)+\frac{\gamma \text{Re}}{20}\frac{\partial }{\partial \bar{r}}\left({\left(1+\left(1-F\right)\varphi \right)}^{2}\bar{r}{\bar{h}}^{3}\right)\\ +\frac{\gamma }{\theta_{0}}\frac{\partial }{\bar{r}\partial \bar{\theta }}\left(\left(1+\left(1-F\right)\varphi \right)\bar{r}\bar{h}\right)=0 \end{array}$$ (10)

where

$$\bar{r}=\frac{r}{r_{0}},\bar{h}=\frac{h}{h_{0}},\bar{\theta }=\frac{\theta }{\theta_{0}},\theta_{0}=\frac{2\pi }{N_{g}},\text{Re}=\frac{\rho \omega h_{0}^{2}}{\mu },\gamma =\frac{6\mu \omega r_{0}^{2}}{\left(p_{a}-p_{c}\right)h_{0}^{2}}$$

## 4. Numerical solution method for dynamic lubrication of micro groove seal

To solve the discrete problem of the first order partial differential, the differential equation shown in Eq (10) can be transformed into the semi discrete integral equation shown in Eqs (11) and (12) by combining the characteristics of the finite volume method [23].


$$\frac{\partial \rho \phi }{\partial t}+\nabla \left(\rho \vec{v}\phi \right)=\nabla \left({ℸ}^{\phi }\nabla \phi \right)+Q^{\phi }.$$ (11)

$$\sum {\left(\rho \vec{v}\phi -{ℸ}^{\phi }\nabla \phi \right)}_{f}\cdot S_{f}=Q_{C}^{\phi }\;V_{C}$$ (12)

The terms in Eq (12) are time term, convection term, diffusion term, and source term in sequence. *ϕ* represents any scalar variable. The equation described in this paper only contains convection and diffusion terms. Therefore, based on the above discretization process, the Reynolds equation should be semi discretized into Eq (13):

$$\sum {\left(\rho \vec{v}\phi -{\vec{V}}^{\phi }\nabla \phi \right)}_{f}S_{f}=0$$ (13)

The steady-state dimensionless Reynolds equation considering cavitation is transformed into a dimensionless vector form as follows

$$\nabla \left\{-\frac{{\bar{h}}^{3}}{12\mu }\nabla F\varphi +\frac{\rho r\omega^{2}h^{3}}{40\mu }\vec{r}+\frac{h\omega r}{2}\vec{\theta }\right\}=0$$ (14)

The gradient of the vector field represented by $-\frac{{\bar{h}}^{3}}{12\mu }\nabla F\varphi +\frac{\rho r\omega^{2}h^{3}}{40\mu }\vec{r}+\frac{h\omega r}{2}\vec{\theta }$ is 0, and the divergence of the gradient is also 0. Similarly, the vector field is a passive field, and the integration within a closed control body is 0, that is

$$\oint \left\{-\frac{{\bar{h}}^{3}}{12\mu }\nabla F\varphi +\frac{\rho r\omega^{2}h^{3}}{40\mu }\vec{r}+\frac{h\omega r}{2}\vec{\theta }\right\}n\text{d}\Gamma =0$$ (15)

where ***n*** is the unit outer normal direction of the control volume boundary, and dΓ is the length of the control volume boundary.

The physical meaning of Eq (15) can be described as the net flow rate of the control body being 0, which means that the mass of the fluid flowing into the control body is equal to the mass flowing out of the control body.

Integrate to obtain the discrete flow integral calculation equation along the given direction:

$$\left\{\begin{array}{c} \;\;{\bar{Q}}^{r}=\int_{\theta_{1}}^{\theta_{2}}\{-{\bar{h}}^{3}\frac{\partial F\varphi }{\partial \bar{r}}+\frac{\gamma Re\bar{r}\bar{h^{3}}{\left(1+\left(1-F\right)\varphi \right)}^{2}}{20}\}\text{d}\theta \\ {\bar{Q}}^{\theta }=\int_{r_{1}}^{r_{2}}\{-{\bar{h}}^{3}\frac{\partial F\varphi }{\theta_{0}^{2}\bar{r}\partial \bar{\theta }}+\frac{\gamma \bar{r}\bar{h}}{\theta_{0}}\left(1+\left(1-F\right)\varphi \right)\}\text{d}r \end{array}\right.$$ (16)

Represented by Eq (16) as follows:

$$Q^{in}-Q^{out}=0$$ (17)

The integral equation mentioned above requires the film thickness value at the seal face, which may have a discontinuity for groove of the sealing ring. Therefore, the value of film thickness in the integral equation may differ due to different mesh densities. To ensure that the numerical model is not dependent on geometric parameters during mesh generation, further processing is required to handle discontinuities.

**Fig 3:**
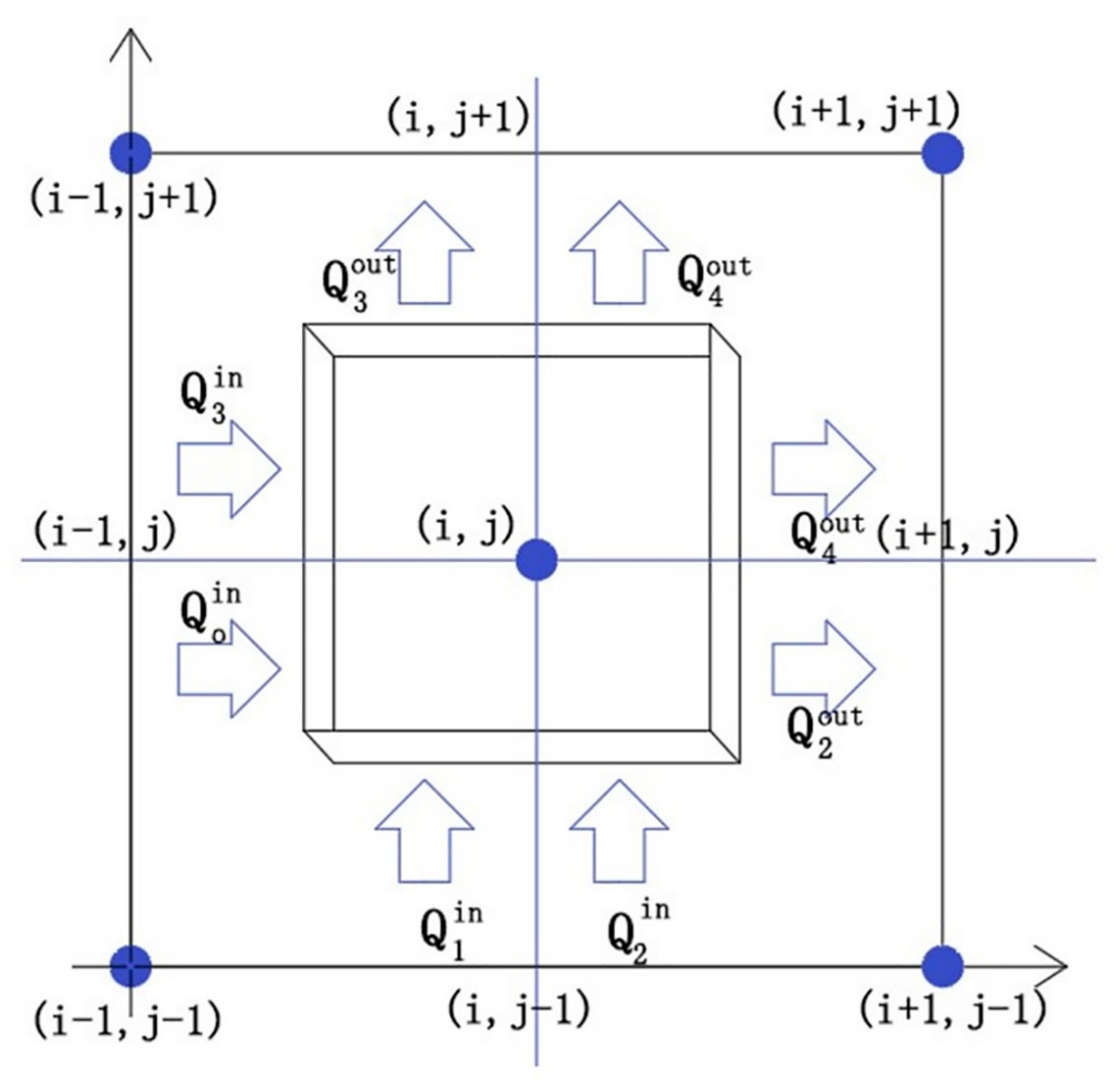
FVM control unit description.

To address the film thickness discontinuity, it is assumed that each control volume is subdivided into four sub-regions, and the film thickness of each sub-region is specified based on the actual film thickness. Considering the unit mass flow in Eq (16) and based on the finite volume method, the following control volumes are selected as shown in Fig 3. Each control volume parameters are concentrated at the center point (*i*, *j*) of the control volume. The center point of each control volume is also a discrete grid node. The control volume is defined as a half-step rectangular region centered on a grid node.

The divergence theorem of the control volume is expressed as

$$Q_{1}^{in}-Q_{2}^{out}+Q_{3}^{in}-Q_{4}^{out}+Q_{1}^{in}+Q_{2}^{in}-Q_{3}^{out}-Q_{4}^{out}=0$$ (18)

For the control body (*i*, *j*), the integral expression is the mass flow rate that flows into or out of the control body for the end face region. In Eq (18), different film thicknesses are selected for integration in different regions.

**Fig 4:**
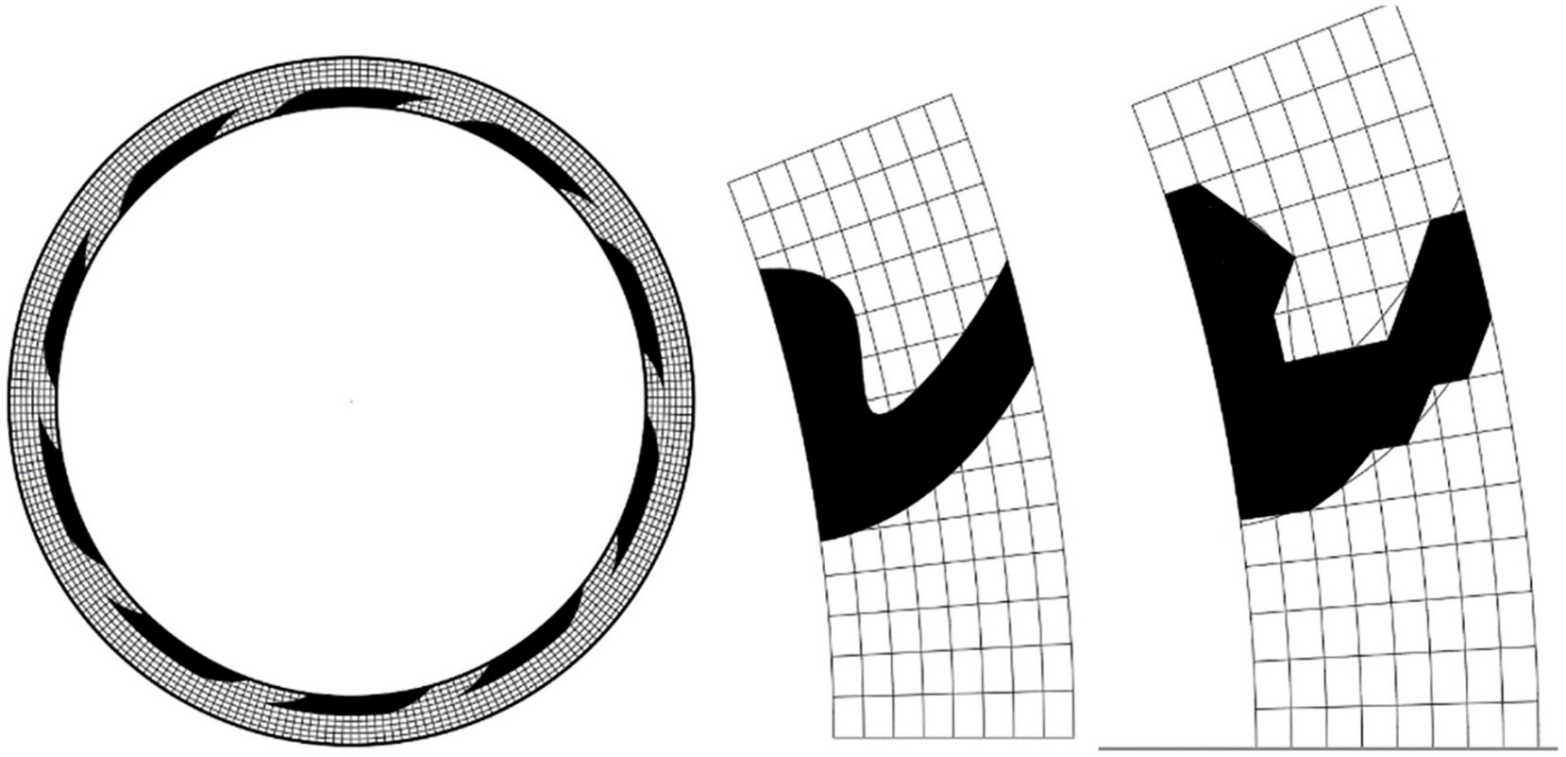
Schematic diagram of groove generation.

For the groove type of the sealing ring shown in Fig 4, a standard orthogonal grid is used for the grid division. If the design of the groove area is generated using spline curves, the groove area and the grid cannot fit perfectly. Therefore, it is considered to further discretize the groove curve and use the nearest adsorption point of each grid line to fit the groove area. The fitting results are shown in Fig 4. The grid of the computational domain is divided along polar coordinates, which is not conducive to computation. Therefore, the use of orthogonal body fitting transformation transforms the computational domain from (*r*, *θ*) to a standard orthogonal coordinate system (*ξ*, *η*).

Taking *Q*_1_^*in*^ as an example for discretization, the derivation process is as follows.


$${\bar{Q}}^{r}=\int_{\theta_{1}}^{\theta_{2}}-{\bar{h}}^{3}\frac{\partial F\varphi }{\partial \bar{r}}+\frac{\gamma Re\bar{r}\bar{h^{3}}{\left(1+\left(1-F\right)\varphi \right)}^{2}}{20}\text{d}\theta$$ (19)

Using second-order central difference to differentiate the partial derivative term in Eq (19):

$$\frac{\partial F\varphi }{\partial \bar{r}}=\frac{F\varphi_{i,j+1}+F\varphi_{i,j-1}-2F\varphi_{i,j}}{\Delta r^{2}}$$ (20)

For the discretization of integrals, assuming a uniform distribution between nodes, Eq (19) can be discretized as

$${\bar{Q}}^{r}=\int_{\theta_{1}}^{\theta_{2}}-{\bar{h}}^{3}\frac{F\varphi_{i,j+1}+F\varphi_{i,j-1}-2F\varphi_{i,j}}{\Delta r^{2}}+\frac{\gamma Re\bar{r}\bar{h^{3}}{\left(1+\left(1-F\right)\varphi \right)}^{2}}{20}\text{d}\theta$$ (21)

According to the coordinate transformation rule, Eq (16) in the (*ξ*, *η*) coordinate system can be derived as

$$\left\{\begin{array}{c} {\bar{Q}}^{\xi }=\int_{\eta_{1}}^{\eta_{2}}\left(-A\frac{\partial F\varphi }{\partial \xi }+B\frac{\partial F\varphi }{\partial \eta }+D\left(1+\left(1-F\right)\varphi \right)+E\right)\text{d}\eta \\ {\bar{Q}}^{\eta }=\int_{\xi_{1}}^{\xi_{2}}\left(B\frac{\partial F\varphi }{\partial \xi }-C\frac{\partial F\varphi }{\partial \eta }+G\left(1+\left(1-F\right)\varphi \right)+H\right)\text{d}\xi \end{array}\right.$$ (22)

where

$$\left\{\begin{array}{l} A=a\frac{{\bar{h}}^{3}}{\left|J\right|},\;B=b\frac{{\bar{h}}^{3}}{\left|J\right|},C=c\frac{{\bar{h}}^{3}}{\left|J\right|}\\ D=-\frac{\gamma \bar{r}\bar{h}}{\theta_{0}}\bar{r_{\eta }},E=\frac{\gamma Re\bar{r}\bar{h^{3}}}{20}\bar{r}\bar{\theta_{\eta }},G=\frac{\gamma \bar{r}\bar{h}}{\theta_{0}}\bar{r_{\xi }},H=-\frac{\gamma Re\bar{r}\bar{h^{3}}}{20}\bar{r}\bar{\theta_{\xi }}\\ a={\left(\bar{r}\bar{\theta_{\eta }}\right)}^{2}+\bar{r_{\eta }^{2}},b=\left(\bar{r}\bar{\theta_{\xi }}\right)\left(\bar{r}\bar{\theta_{\eta }}\right)+\bar{r_{\xi }}\bar{r_{\eta }}\\ c={\left(\bar{r}\bar{\theta_{\xi }}\right)}^{2}+\bar{r_{\xi }^{2}},\left|J\right|=\bar{r_{\xi }}\left(\bar{r}\bar{\theta_{\eta }}\right)-\bar{r_{\eta }}\left(\bar{r}\bar{\theta_{\xi }}\right) \end{array}\right.$$ (23)

The body fitted coordinate transformation only converts the computational domain mesh, and the discretization process remains unchanged using the finite volume method.

Perform the above discretization process on eight nodes within a control body, and the final form is as follows.

$$\begin{array}{c} a_{0}\varphi_{i,j}+a_{1}\varphi_{i-1,j}+a_{2}\varphi_{i+1,j}+a_{3}\varphi_{i,j-1}+a_{4}\varphi_{i,j+1}+a_{5}\varphi_{i-1,j-1}+\\ a_{6}\varphi_{i+1,j-1}+a_{7}\varphi_{i-1,j+1}+a_{8}\varphi_{i+1,j+1}+a_{9}=0 \end{array}$$ (24)

where

$$\left\{\begin{array}{l} a_{0}=\;-3\left(A_{1}+A_{2}+A_{3}+A_{4}+C_{1}+C_{2}+C_{3}+C_{4}\right)F_{i,j}+4\left(B_{1}-B_{2}-B_{3}+B_{4}\right)F_{i,j}\\ +3\left(-D_{2}-D_{4}-G_{3}-G_{4}\right)\left(1-F_{i,j}\right)\\ a_{1}=\left[3\left(A_{1}+A_{3}\right)-C_{1}-C_{3}\right]F_{i-1,j}+\left(3D_{1}+3D_{3}-G_{3}\right)\left(1-F_{i-1,j}\right)\\ a_{2}=\left[3\left(A_{2}+A_{4}\right)-C_{2}-C_{4}\right]F_{i,j-1}-G_{4}\left(1-F_{i+1,j}\right)\\ a_{3}=\left[-A1-A2+3\left(C1+C2\right)\right]F_{i,j-1}+\left(-D_{2}+3G_{1}+3G_{2}\right)\left(1-F_{i,j-1}\right)\\ a_{4}=\left[-A3-A4+3\left(C3+C4\right)\right]F_{i,j+1}-D_{4}\left(1-F_{i,j+1}\right)\\ a_{5}=\left[A1+C1-4B1\right]F_{i-1,j-1}+\left(D_{1}+G_{1}\right)\left(1-F_{i-1,j-1}\right)\\ a_{6}=\left[A2+C3+4B3\right]F_{i+1,j-1}+G_{2}\left(1-F_{i+1,j-1}\right)\\ a_{7}=\left[A3+C4-4B4\right]F_{i-1,j+1},D_{3}\left(1-F_{i-1,j+1}\right)\\ a_{8}=\left[A4+C4-4B4\right]F_{i+1,j+1}\\ a_{9}=4\left(D1+E1-D2-E2+D3+E3-D4-E4\right)\text{+}\\ 4\left(G1+H1+G2+H2-G3-H3-G4-H4\right) \end{array}\right.$$ (25)

Assuming the number of grids divided is m×n, the numerical equation is a system of (m-1) × (n-2) linear equations. The iterative matrix can be obtained.

AX=B (26)

$$\left\{\begin{array}{c} A=\left[\begin{array}{ccc} \vec{a_{0}} & \ldots & \vec{a_{8}} \end{array}\right]\\ X=\left[\begin{array}{c} p_{i,j}\\ \vdots \\ p_{i+1,j+1} \end{array}\right]\\ B=\vec{a_{9}} \end{array}\right.$$ (27)

where $\vec{a_{i}}$ represents the vertical expansion of the matrix.

Define convergence conditions:

$$\overset{m}{\underset{i=1}{\sum }}\overset{n-1}{\underset{j=1}{\sum }}\frac{\left|p_{\text{i},\text{j}}^{k+1}-p_{i,j}^{k}\right|}{p_{i,j}^{k}}<10^{-6}$$ (28)

By iterating until the convergence condition is met, the pressure distribution in the calculation area can be obtained. Opening force *F*_0_ of the liquid film is as follows.


$$F_{0}=\int_{r_{1}}^{r_{0}}\int_{0}^{2\pi }pr\text{d}\theta \text{d}r$$ (29)

The validity of the numerical method for general groove seal established in this paper is verified by analyzing the performance of seal device mentioned in [24]. And compare was made with the calculation result based on shallow groove theory.

**Fig 5:**
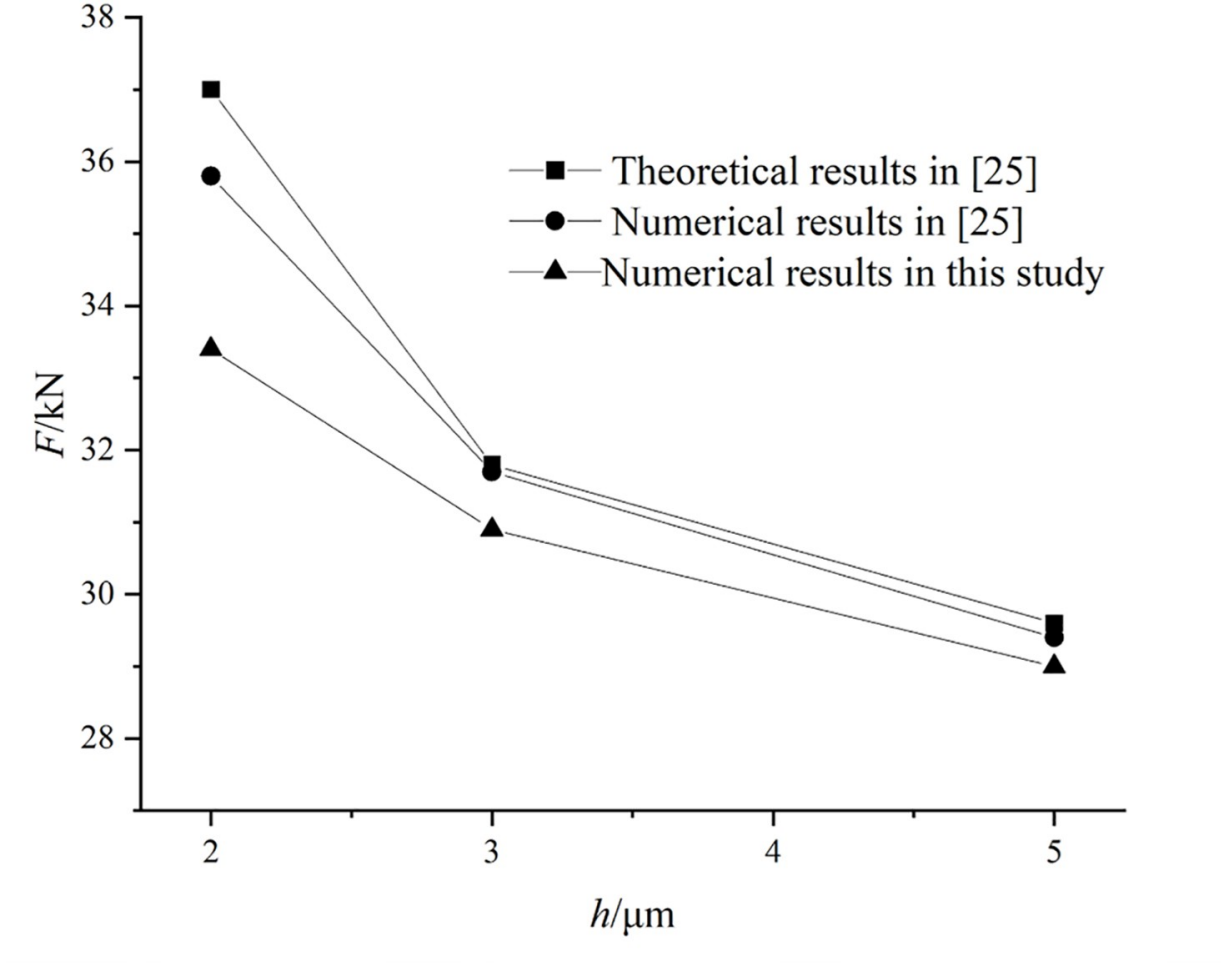
Comparative results with different calculations.

As is shown in Fig 5, the comparative results for load carrying capacity are obtained. With the minimum film thickness *h* decreases, load carrying capacity *F*_0_ decline. The numerical results in this study match well with theoretical and numerical ones in Ref. [24], verifying the accuracy of novel numerical model for general groove seal. However, we find that the values obtained from the presented method are smaller than those of the published results. One important reason is that this paper uses the improved JFO cavitation boundary condition to simulate the cavitation phenomenon in seal gap flow. The cavitation effect greatly reduces the hydrodynamic pressure effect under low pressure conditions. A certain size of cavitation zone lowers the pressure distribution of the entire lubrication zone, which leads to a smaller bearing capacity.

## 5. Comparison of sealing performance of typical grooves

This section focuses on comparisons of sealing performance of typical grooves of the sealing ring. We analyze the sealing performances with different groove shapes under the same working condition and structural parameters of the same sealing ring through the numerical method in Section 3. The working conditions are as follow. The minimum fluid film thickness *h*_0_ = 10 μm, shaft rotating speed *n*_*s*_ = 2000 rpm, inner radius *r*_1_ = 58.6 mm, outer radius *r*_2_ = 65 mm. The angle occupied by each computational domain is *β* = 2π/*N*_*g*_. The outlet pressure in the outer diameter is equal to atmospheric pressure, that is *p*_*a*_ = *p*_0_ = 0.1×10^6^ Pa, and the inlet pressure in the inner diameter *p*_1_ = 1×10^6^ Pa. The periodic boundary condition is *p*(0) = *p*(*β*).

### 5.1 Rectangular groove

**Fig 6:**
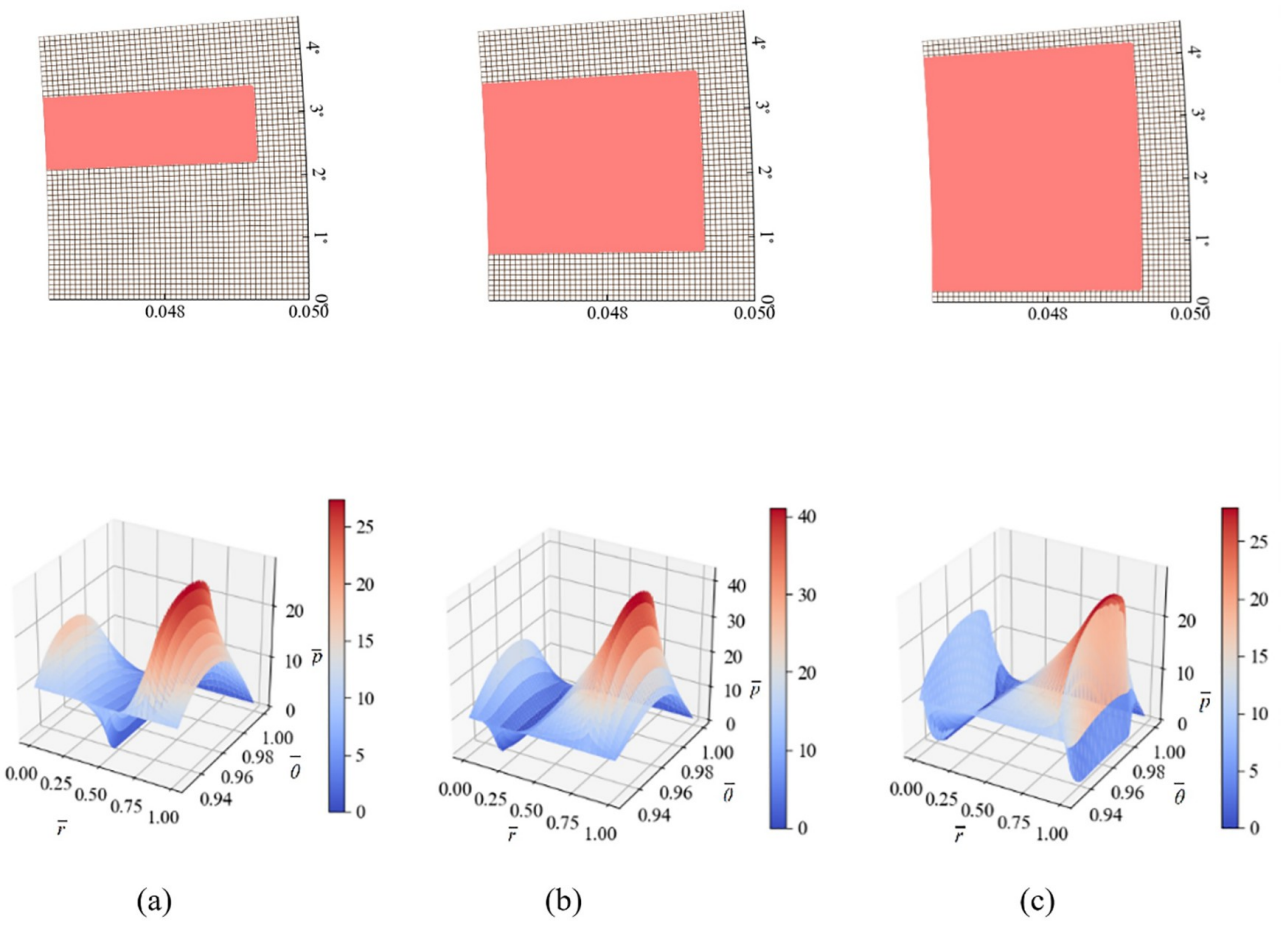
Three rectangular groove shapes with different axial widths and their corresponding pressure distributions.

As a simple type of groove in terms of structure, rectangular groove has been widely used due to its simple structure and convenient processing. The specific shape of the rectangular groove can be controlled by two parameters, namely the length and width of the groove. In this section, three typical rectangular grooves shown in Fig 6 are selected for numerical calculations, and the pressure distributions of the rectangular grooves are obtained.

By observing the overall pressure distribution of the three rectangular grooves in Fig 6, the bearing capacity of the three groove types is 45.22 kN, 53.42 kN, and 37.81 kN, respectively. From the trend of pressure distribution changes, it can be seen that as the width of the rectangular groove increases, the bearing capacity of the sealing ring first increases and then decreases with the width of the rectangular groove. For the two groove types in Fig 6(a) and 6(b), the trends of their pressure distributions are basically consistent, but the peak pressure distribution in Fig 6(b) is obviously higher than that in Fig 6(a). Therefore, it can be considered that with the increase of the width of the rectangular groove, the peak pressure of the sealing ring further increases, which is caused by the more fully exerted dynamic pressure effect. For Fig 6(b) and 6(c), as the width of the rectangular groove further increases, the peak pressure starts to decrease. This is because the dynamic pressure effect cannot be fully exerted due to the narrow non-groove area.

When the width of the rectangular groove is appropriate, geometric shapes can more effectively utilize the fluid dynamic pressure effect. In this way, the fluid dynamic pressure reaches its highest pressure, and the range of high-pressure areas also significantly increases, thereby greatly enhancing the fluid carrying capacity. The arrangement of the groove not only enhances the fluid dynamic pressure effect in the convergent region of the groove edge, but also effectively suppresses the cavitation effect in the divergent region of the seal groove boundary, thereby improving the overall fluid carrying capacity of the seal surface.

**Fig 7:**
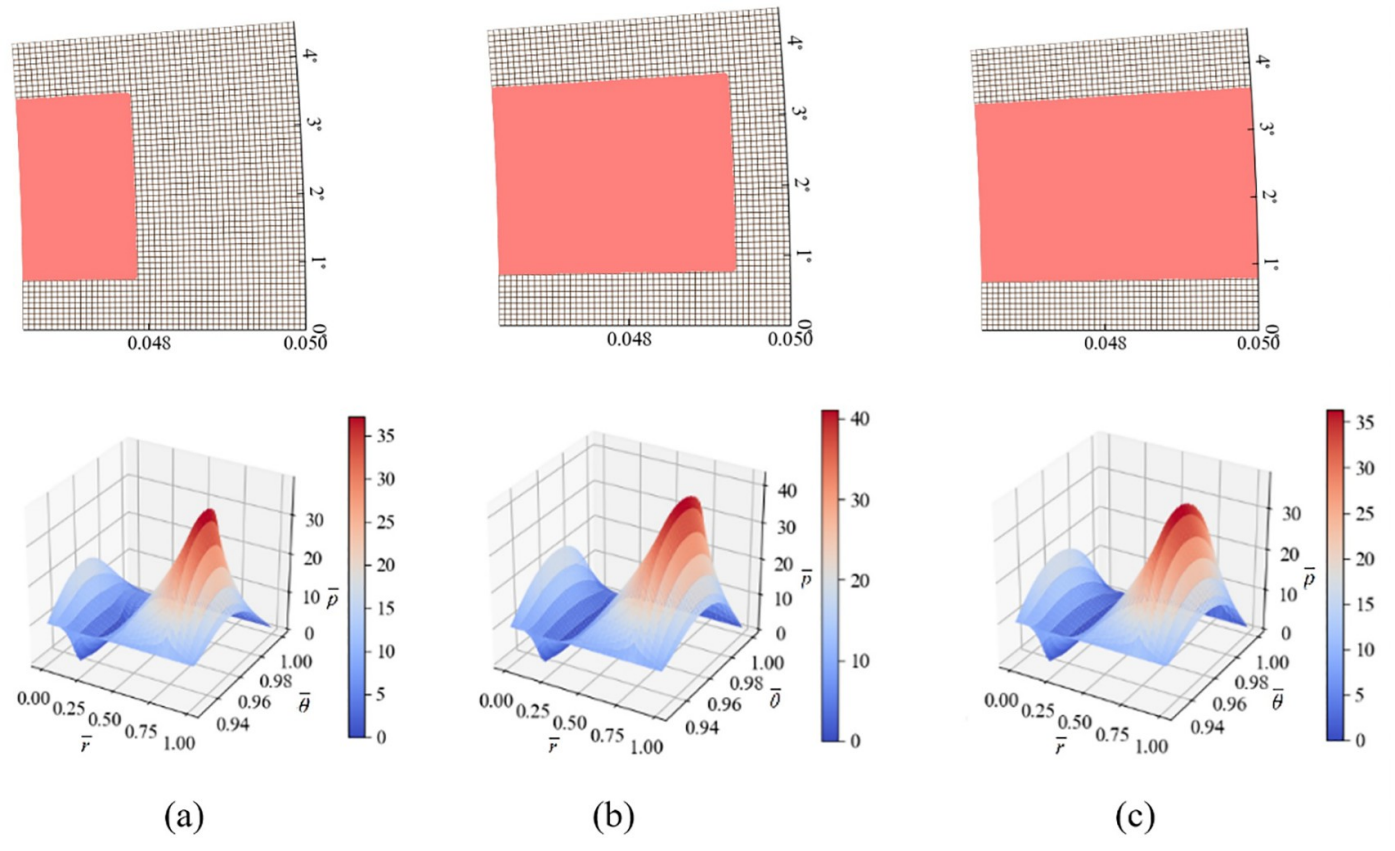
Three rectangular groove shapes with different radial lengths and their corresponding pressure distributions.

Fig 7 shows three rectangular grooves with varying groove lengths. The bearing capacities of the three groove types are 30.57 kN, 52.08 kN, and 43.63 kN, respectively. It can be found that with the increase of the groove length, the bearing capacity of the sealing ring significantly increases. However, when the groove length is too long to become a through groove, the bearing capacity decreases, and the through groove causes excessive oil leakage.

Increasing the outer diameter dimensions of the rectangular groove leads to a stronger fluid carrying capacity per unit area. This is because the seal has a larger circumferential dimension, which allows for more effective utilization of the fluid dynamic pressure effect along the circumferential step. The greater the radial groove dam ratio, the stronger the fluid dynamic pressure effect in the seal micro-groove. The fluid dynamic pressure effect forms at the converging boundary of the film thickness on the groove edge. The more boundaries the groove edge has, the stronger the fluid carrying capacity of the surface. However, it is important to note that the radial groove dam ratio should not be too large for the sake of sealing efficiency in the seal system. Otherwise, the micro-groove will radial join the inner and outer fluid passages, resulting in a loss of sealing effectiveness.

Based on the analysis in this section, it can be determined that the performance of the rectangular groove varies significantly with the groove length and width. The optimal rectangular groove structure is similar to the one shown in Fig 7(b), which can better utilize the hydrodynamic lubrication effect and has a higher bearing capacity.

### 5.2 Spiral groove

**Fig 8:**
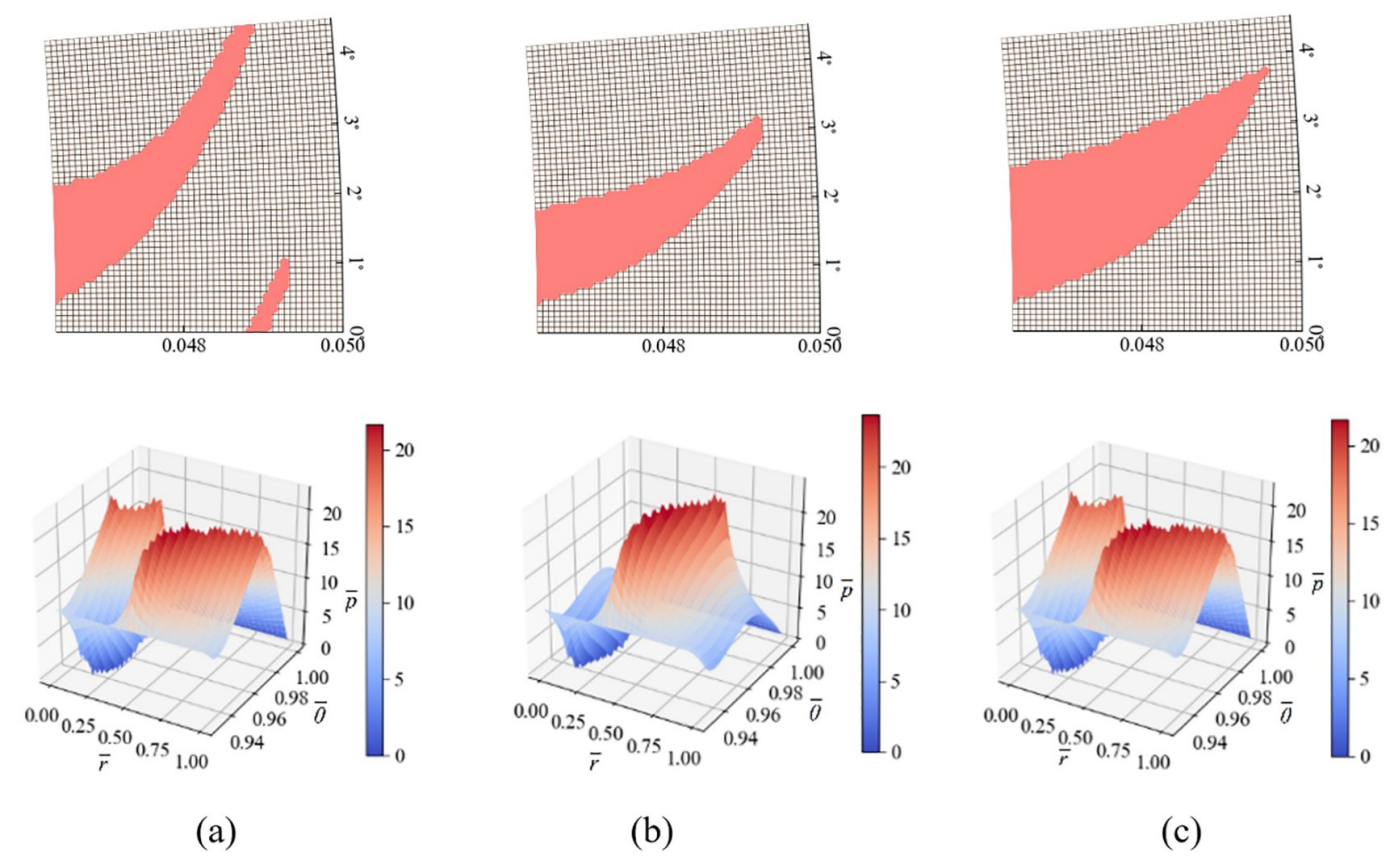
Several spiral grooves and their corresponding pressure distribution.

Based on the different selected helix lines, there can be many variations of the spiral groove. In this section, we mainly consider two factors of the spiral groove, namely the span of the groove across the period and the ratio of the groove area. Three types of grooves as shown in Fig 8 are designed, where Fig 8(a) represents a groove with a span across the period, and its area ratio is consistent with Fig 8(b). The spiral groove in Fig 8(c) has a larger area ratio. The pressure distribution results of the three types of spiral grooves are shown in Fig 8, and the bearing capacities of the three spiral grooves are 38.51 kN, 60.57 kN, and 40.38 kN, respectively. From the calculation results, it can be seen that the spiral groove in Fig 8(b) has better bearing performance. By observing the pressure distribution, it can be found that the peak pressure of the three types of grooves is similar, but the high-pressure zone in Fig 8(b) is more evenly distributed, which can maintain a longer high-pressure zone due to the effect of the helix line and better utilize the hydrodynamic lubrication effect.

At the converging boundary of the fluid film on the groove edge of the spiral groove, a high-pressure region is formed due to the "wedge effect". The structural form of the groove profile not only effectively utilizes the fluid dynamic pressure effect in the converging gap, but also reduces the impact of cavitation in the diverging gap. This reflects the superior fluid dynamic pressure effect of the groove shape design, which enables the seal to transition more quickly from a mixed lubrication state to a fully fluid-film hydrodynamic lubrication state.

Through the above analysis and comparison, we have found the following conclusions.

1. By observing the pressure distribution of different groove types, it is easy to find that the high-pressure zone and low-pressure zone of the groove mainly occur at the non-groove boundary of the groove area. The phenomenon of wider high-pressure zone distribution in the spiral groove proves that increasing the groove length can effectively improve the range of the high-pressure zone, but it will not increase the peak value of the high-pressure zone, and may even decrease.
2. If the groove line is too long, as shown in Fig 8(a), it will cause a rapid decrease in peak pressure. This is because the long groove line occupies a long circumferential distance, resulting in a narrowing of the circumferential width of the groove, and the fluid flow along the circumferential distance is too short, resulting in the inability to effectively form the hydrodynamic lubrication effect.
3. As shown in Fig 7, if the given cycloid radius is sufficient and the circumferential width is wide enough, increasing the groove length can effectively improve the bearing capacity in order to produce sufficient the hydrodynamic lubrication effect within a period.

## 6. Conclusions

Specifically for the calculation of flow fields in sealing rings with micro-grooves in the pharmaceutical kettles, a numerical model is established to accurately describe the flow field using the Reynolds equation. By considering the principle of mass conservation, the numerical form without considering the membrane thickness derivative term is derived. Coordinate transformation is applied to adapt to the curved shape of the seal groove. The numerical model is used to calculate the pressure distribution in the flow field. The model takes into account the periodic characteristics of the groove shape of the sealing ring and introduces periodic boundary conditions. It also considers the possibility of cavitation in the flow field and modifies the Reynolds equation by introducing the JFO cavitation model. For the issue of membrane thickness discontinuity, the mean-weighted approach is used to handle the membrane thickness. To account for the diversity of groove shapes, an approximation method using node adhesion is presented. The model is built based on the physical background of mass conservation and can adapt to any different groove shapes, demonstrating strong versatility.

This simulation model is employed to calculate the evaluation indicators of different seal groove shapes. Regarding the analysis to different groove types, the performance of seal groove types under different parameters is calculated to analyze the relationship between sealing performance and groove type parameters of rectangular grooves and spiral grooves. These grooves on the end face of the seal can satisfy the conditions for generating the hydrodynamic lubrication effect. The general reasons for the well-performed groove types are analyzed, and three conditions for good sealing performances based on simulations are proposed. This helps to the design of the seal groove for the seals in the pharmaceutical kettles.

The seal may have an opening force provided through both the oil film and the rough asperities. In subsequent researches, a more general mechanical model should be established to calculate the actual oil film bearing capacity of the seal.
